# Determinants of underweight, stunting and wasting among schoolchildren

**DOI:** 10.1186/s12889-014-1337-2

**Published:** 2015-01-17

**Authors:** Mekides Wolde, Yifru Berhan, Alemzewed Chala

**Affiliations:** Hawassa University, College of Agriculture, Hawassa, Ethiopia; Hawassa University, College of Medicine and Health Sciences, Hawassa, Ethiopia

**Keywords:** Crossectional, Ethiopia, Determinant, Schoolchildren, *Under*-*nutrition*

## Abstract

**Background:**

The cause of under-nutrition in schoolchildren is complex and varying from region to region. However, identifying the cause is the basic step for nutritional intervention programs.

**Methods:**

School based cross-sectional survey was conducted among 450 schoolchildren aged 7-14 years, using multi-stage sampling techniques in Dale Woreda, southern Ethiopia.

A structured questionnaire and 24-hour recall methods were administered to determine the sociodemographic and dietary intake of participants. Stool microscopic examination was done. Weight and height were measured using a standard calibrated scale. Odds ratio generated from logistic regression was used to determine the strength of variables association.

**Results:**

Older age group (10-14 vs. 7-9) (AOR = 3.4; 95% CI, 1.7-6.6) and having Trichuris Trichura infection (AOR = 3.9; 95% CI, 1.4 -11.6) increased the risk of being stunted. Children whose mothers have completed primary education are less likely to be stunted than children whose mothers do not have formal education (AOR = 0.3; 95% CI, 0.2-0.8).

Having large family size (AOR = 3.3; 95% CI, 1.4-7.9) and inadequate intake of carbohydrate (AOR = 3.1; 95% CI, 1.4-6.8) were independent predictors of wasting. Children whose mothers completed primary education are less likely to be underweight (AOR = 0.3; 95% CI, 0.1-0.9). Children live in food insecure households are more likely to be stunted, under-weight and wasted than children live in food secure households (AOR = 2.5; 95%, 1-5.6; AOR = 3.9; 95% CI, 1.2-12.0; AOR = 4.8; 95% CI, 1.7-13.6;).

**Conclusion:**

Household food insecurity, low maternal education and infection with Trichuris trichura were some of the major factors contributing to under-nutrition in the study area.

## Background

Under- nutrition among school-age children is a common problem in developing nations. It may turn out from a broad range of aspects like prenatal under-nutrition, deficiencies of macro and micronutrient, infection and possibly socioeconomic conditions [1].

United nation for children’s fund [2] reported that more than 200 million school-age children were stunted by the year 2000.In the same report, it was pointed out that the proportion of stunted schoolchildren with impaired physical and mental development will grow up to 1 billion by the year 2020 unless a tangible action is undertaken.

The economic cost of under-nutrition is highly substantial. According to the World Health Organization, underweight is the single largest risk factor contributing to the global burden of disease in the developing world. It leads to nearly 15 percent of the total disability-adjusted life years (DALY) losses in countries with high children mortality [3]. It is also proved that 1% loss in adult height occurred due to childhood stunting which in return associate with 1.4% loss in productivity [4]. Moreover; poor nutrition and health among children contribute to the general inefficiency of education systems worldwide. Varies researches have shown that improved nutrition and health lead to better performance, fewer repeated classes and reduced dropout rates [5].

In Ethiopia, particularly in the study area, there is scarcity of information on the determinants of under-nutrition among schoolchildren. Moreover, schoolchildren are at high risk of nutritional deficiency and their nutritional status is poorly documented. Because most of the studies in this country have been conducted on under five children. We surmise that identifying the contributing factors for under-nutrition among schoolchildren is the basic step to set a sustainable and effective nutritional intervention in the study area. Thus, the present study was designed to assess determinants of under-nutrition among schoolchildren aged 7-14 years.

## Methods

This school based cross-sectional survey was conducted among randomly selected schoolchildren aged 7-14 years old from randomly selected three schools in Dale

Woreda. Dale woreda is found in the Sidama zone of Southern Nations and Nationalitities Peoples’ Regional State of Ethiopia. The woreda is located about 326 Km South from Addis Ababa, capital city of Ethiopia. The Woreda has a total area of 28,444 hectares; total population of 222,068 with 37,027 households. The Woreda is characterized by 1% high land and 99% mid-altitude agro-ecologies and produces a variety of crops and livestock. The area is known for its coffee production. At the time of the research, the Woreda had 36 kebeles, 7 health centers, 29 health posts and 1 hospital.

The study was conducted at three schools in October 2012 and 450 students were sampled using single population proportion formula:

$$ \mathrm{n}=\left[\frac{{\left({\mathrm{Z}}_1-\frac{\upalpha}{2}\right)}^2\uprho \left(1-\uprho \right)}{{\mathrm{d}}_2}\right] $$

Where: Z = Standard normal variable at 95% confidence level (1.96)

P = Anticipated proportion, (23.1%)

d = 0.05 (5% margin of error)

To minimize errors arising from the likelihood of non-compliance, 10% of the sample size was added. Finally, a design effect (DE) of 1.5 is used to minimize bias arising from not using simple random sampling technique.

**Figure 1:**
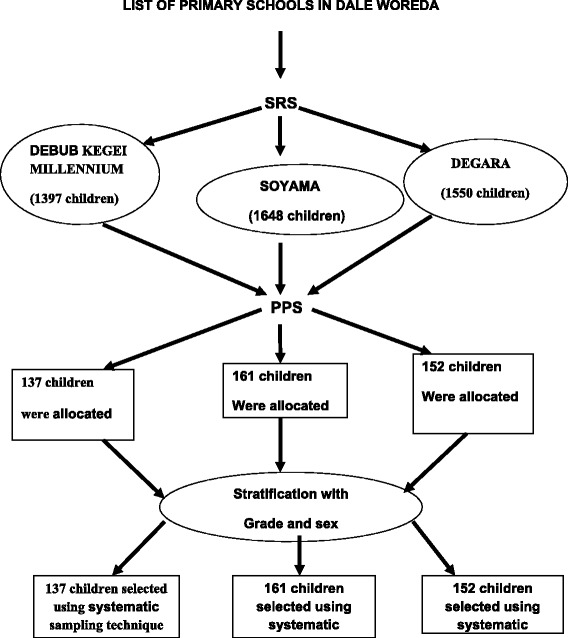
**Schematic presentation of sampling procedure, Dale Woreda, 2012.**

A multi-stage sampling technique was used to select a representative sample of schoolchildren from the study area. The list of schools existing in Dale Woreda was obtained from Woreda’s education office, and three schools (Debub Kegie Millennium, Soyama and Degara) were selected using lottery method. List of all students aged 7-14 years and whose grade 1-8 was obtained from each school head. A total of 1397, 1648 and 1550 schoolchildren were found in Debub kegie millennium, Soyama and Degara schools, respectively. The number of schoolchildren aged 7-14 years from each school and grade included in the study was determined by using proportional allocation (PPS) to size. Finally, systematic sampling system was applied to select the study participants from each grade and sex using the respective class rosters for 2011/2012 academic year as the sample frame; the sampling interval for the systematic sampling system was calculated by dividing the total number of students in the roster to the specific students to be selected from each class (Figure 1).

A standardized questionnaire was developed based on known and hypothesized risk factors. Household food insecurity status was assessed using the nine Food and Nutrition Technical Assistance (FANTA) scale guideline questions [6]. The questionnaire was constructed in English and translated into Sidama (local language). The questionnaire was pretested among 45 schoolchildren (10% of the sample size). The socio-demographic and dietary interviews were done at the household level.

Parents and children whose age is 8 years and above were respondents. An oriented health extension worker recorded observations on physical situations for each child in the school setting. The data collectors all had education levels of grade 10 or above and were native to the study area.

After data collection had been completed, some of the variables were categorized for analysis purpose. Categorized variables include: age of a child which is grouped into two (7-9 years and 10 and above years), family size grouped into three: small, medium and large, maternal educational status grouped into three: no formal education, primary education completed and secondary education and above, household food insecurity status grouped into four: food secure, mild food insecure, moderate food insecure and severe food insecure households.

For the anthropometric data digital portable calibrated SECA weighing scale was used to measure the weight of studied children. The children were weighed wearing lightly clothed, without shoes and with empty pockets. The calibrated SECA balance scale has intervals/sensitivity of 0.1 kg with a capacity of 130 kg.

Height was measured to the nearest 0.1 cm precision and length up to 2 meters using the same device that has a scale and sliding headpiece. Weighing scale was calibrated to zero before taking every measurement. Clinical examination of study participants was done for variables such as the presence or absence of clean clothing, dental caries, trimmed fingernails, presence of dirt on fingernails, wearing shoes, paleness on the conjunctiva and palms, edema and for any more body abnormalities by the principal investigators and an assistant nurse. All the observations were recorded in the recording format.

Stool samples were collected and preserved using WHO standard operating procedures for the parasitological examination of feces [7]. Accordingly, study participants provided labeled plastic cups with serial numbers, soft tissue paper, and clean wooden applicator stick. And they were instructed to bring 2 g or about a thumb of fresh stool sample of their own. Students who brought inadequate stool sample were re-instructed to bring another sample. Samples collected from study participants were preserved in a tube containing 10% formalin in 0.85% saline, and were transported to Hawassa university microbiology laboratory for microscopic examination following WHO standard operating procedures [7]. Ten percent (10%) of the total fecal specimen (450) was re-checked in the laboratory by a senior laboratory technologist.

Dietary assessments were conducted at the household level and the respondents were children’s parents and children themselves. Trained data collectors filled the 24 hour recall dietary history with specific probes, to help the respondent remember all foods consumed throughout the day. To account for any day of the week effects on food and/or nutrient intake, weekends, weekdays and market days were proportionately represented in the survey.

Ethical clearance for this study was obtained from the institutional review board of Hawassa University. We obtained a written informed parental consent. The students’ privacy during the interview, stool collection, and anthropometric measurements were maintained and data obtained from them was kept confidential.

All statistical analyses were done using SPSS for windows version 16 statistical package. Data entry for anthropometric indices was made using WHO Anthro plus version 1.0.4. The nutrient and energy content of foods consumed by the index child in the preceding 24-hours was calculated using the Ethiopian food composition table [8] and micro soft excel.

All continuous data were checked for normality using the kolmogorov-smirnove test. Descriptive statistical tests were applied to indicate the prevalence of under-nutrition as frequencies and percentages. To test the presence as well as strength of association between under nutrition and factors, binary and multivariate logistic regression tests were used. Variables used for the regression model include: sex of a child, age of a child, maternal educational status, family size, family monthly income, infection with ascariasis only, hookworm only, trichuriasis only, household food security status, and dietary intake interms of energy, carbohydrate, protein, Fe, Zn and Vit A.

Factors showed significant association in the univariate analysis and factors identified in different literatures as predictors were entered into the multivariate analysis.

Under-nutrition was defined for a child, who had less than-2 z-scores (SD) from the NCHS median reference population values [9].

### Operational definitions

**Adequate intake**: intake of the individual is equal or above the estimated average intake (EAR) for nutrients which have EAR or intake equal or above the recommended daily allowances (RDA) for nutrients which do not have EAR.

**Helminthes**: are parasites which are multicellular, bilaterally symmetrical worms having three germ layers, transmitted through contact with fecally contaminated soil or water.

**Helminthic infections positive**: direct microscopic evidence of one or more helminthic parasites.

**Personal hygiene indicators**: These indicators are: responses of participants to questions such as hand washing practices before meal, hand washing practices after use of latrine, trimmed finger nails, neatness of clothing, use of detergent to wash hands and frequency of body bath were considered. Score 0 for no practice, 1 for some times and 2 for mostly practiced.

## Results

### Socio-demographic characteristics

Out of the total sample, 445 (98.8%) were eligible for analysis. The mean (SD) age of the study participants was 10.7 (±2.0) years. The majority of this study participants, 425 (95.5%) and 442 (99.3%), were protestant and Sidama by religion and ethnicity, respectively. Farming was the main source of economy for approximately 90% of the households. Two hundred thirty five (52.8%) and 100(22.5%) of the study participants’ mothers and fathers had never attended a formal education, respectively. The studied households had median family size of six. Most of the households, 426 (95%), were headed by the father. Two hundred fifty two (56.6%) of the participants reported a family monthly income of about 10.0 USD or below per month. The access to mass media was 44.7% (Table 1).

**Table 1:** **Socio-demographic characteristics of studied children in the age range of 7-14 years in Sothern Ethiopia**

**Variable**	**No (%)**
***Age, mean(SD)***	10.7 (±2)
***Sex***	
Male	209(47%)
Female	236 (53%)
***Ethnicity***	
Sidama	442(99.3)
Wolayita	3 (0.7)
***Religion***	
Protestant	425(95.5)
Muslim	9(2)
Catholic	7(1.5)
Orthodox	4(0.9)
***Family size***	
1-5	136(30.6)
6-7	208(46.7)
8-11	101(22.7)
***Maternal education***	
No formal education	235(52.8)
Read and write	124(27.9)
Primary education	78(17.5)
Secondary and above	8(1.8)
***Paternal education***	
No formal education	100(22.5)
Read and write	146(32.8)
Primary education	144(32.4)
Secondary and above	55(12.4)
***Monthly income, USD***	
≤10	252(56.6)
11-25	133(29.9)
>25	60(13.48)
***Occupation of the head***	
Farmer	400(89.9)
Government employee	30(6.7)
Merchant	13(2.9)
Unemployed	2(0.4)
***Household food security status***	
Food secured	242(54.4)
Mild food insecure	129(29)
Moderately food insecure	29(6.5)
Severely food insecure	45(10.1)
***Mean nutrient intake mean(SD)***	
Mean Energy intake	2275.0
Mean protein intake	57.2
Mean CHO intake	286.1
***Personal hygiene score***	
Very good	152(34.2)
Good	241(54.2)
Poor	52(11.7)

### Water, environmental and personal hygiene conditions

Tap water was the main source of drinking water for approximately half of the studied households, 234 (52.6%). Three hundred fifty-nine (80.7%) of the respondents never used a treatment for their drinking water.

Two hundred thirty eight (53.5%) had a pit latrine with slab. It was also found that 180 (40.4%) of the households disposed their solid wastes in an open field.

This study has revealed that 182 (40.9%) of the respondents used soap or ash most of the time to wash their hands. One hundred ninety four (56.4%) of the studied children had trimmed their fingernails, but 130 (29.2%) did not wash their hands before meals. It was also observed that 139 (31.2%) of the participants did not wear shoes on the date of data collection.

### Under nutrition

Anthropometric measurements of weight and height of children were done. Accordingly, stunting was found to be a common nutritional problem, in which 25.6% of studied children were found to be below-2SD while 10.3% of the children were severely stunted. Based on the BMI for age status, 64 (14%) of studied children had wasting while, forty two (19%) of studied children had underweight (Table 2).

**Table 2:** **Prevalence of under nutrition of studied children aged 7-14 years (HAZ and BMI) and aged 7-9 years (WAZ) in Southern Ethiopia**

**Variable**	**No (%)**	**Mean (SD)**
***Height for age (HAZ)***		
** Below -3SD (Severe)**	46(10.2)	
** -3 SD to -2.01SD (moderate)**	68(15.1)	
** Below -2SD (moderate and severe)**	114(25.3)	
** Mean Z score (SD)**		-0.47(2.1)
** -2SD and above(non-stunted)**	336(74.7)	
***BMI***		
** Below -3SD (Severe wasting)**	8 (1.8)	
** -3.00SD to -2.01SD (moderate)**	56(12.4)	
** Below -2SD (moderate and severe)**	64 (14.2)	
** Mean Z score (SD)**		-0.66(1.2)
** -2SD to +1 SD (not wasted)**	342(76)	
** Above + 1SD (over weight/obese)**	33(7.4)	
** Above + 2 SD (Obesity)**	11(2.5)	
***Weight for age (WAZ)***		
** Below -3SD (Severe underweight)**	9(4.1)	
** -3SD to -2.01SD (moderate)**	33(15.2)	
** Below -2 SD (underweight)**	42(19.3)	
** Mean Z score (SD)**		-0.39(1.5)
** -2SD to +1SD (not underweight)**	136(62.3)	
** Above + 1SD (over weight/obese)**	30(13.8)	
** Above + 2 SD (Obesity)**	10(4.6)	

Stunted children include both moderately and severely stunted children. Wasted children are the sum of moderately and severely wasted children. Underweight children also include the sum of moderately and severely underweight children.

### Intestinal Helminthiasis

The overall prevalence of helminthes infection was 286(64.3%) while soil transmitted helminthes infections namely: Ascaris lumbericoides, Hookworm and Trichuris trichiura infection were more common in the studied children. Two hundred fifty six (57.6%) of children were infected by Ascaris lumbericoides. Infection with more than one helminthes (mixed infection) was found in 33 (7.4%) of studied children.

### Determinants of underweight, stunting and wasting

Binary logistic regression was carried out to determine factors associated with under-nutrition. Infection with Trichuris trichura, sociodemographic characteristics, dietary intake and household food insecurity status were major factors associated with under-nutrition.

According to the binary logistic regression model maternal education status (AOR = 0.3; 95% CI, 0.1-0.9) and household food insecurity (AOR = 3.9; 95% CI, 1.2-12) were independently associated with the child’s low weight for age status (Table 3). Multivariate analysis of risk factors for stunting revealed that the odds of being stunted increased 4 times for children who had Trichuris trichura infection than children who do not have infection.

**Table 3:** **Multivariate analyses of risk factors for under-weight in Southern Ethiopia**

**Variable**	**Normal Weight**	**Under Weight**	**Crude OR (95% CI)**	**Adjusted OR(95% CI)**
***Sex***				
Male	86	27	1	1
Female	90	15	0.5(0.3,1.1)	0.5(0.2,1.2)
***Age, years***				
7-9	105	17	1	1
10-14	71	25	2.2(1.0,4.3)	1.8(0.8,4.1)
***Family size***				
Small	67	15	1	1
Medium	82	16	0.9(0.4, 1.9)	0.7(0.3,1.7)
Large	27	11	1.8(0.7,4.5)	1.5(0.5,4.6)
***Maternal education***				
No formal education	80	31	1	1
Read and write	51	5	0.3(0.1,0.7)	0.3(0.1,0.9)**
Primary education	41	6	0.4(0.1,0.9)	0.4(0.1,1.3)
Secondary and above	4	0	NA	NA
***Monthly income, USD***				
<10	96	29	1	1
11-25	53	8	0.5(0.2,1.2)	0.5(0.2,1.4)
>25	27	5	0.6(0.2,1.7)	0.5(0.1,1.8)
***Personal hygiene***				
Very good	21	2	1	1
Good	115	17	0.9(0.4,2.3)	1.2(0.2,6.2)
Poor	40	23	1.6(0.6,4.1)	3.8(0.7,2)
***Food insecurity status***				
Food secure	112	15	1	1
Mild food insecure	38	15	2.9(1.3,6.6)	2.4(0.9,6.2)**
Moderate food insecure	7	2	2.1(0.4,11.0)	2.7(0.4,20)
Severe food insecure	19	10	3.9(1.5,10.0)	3.9(1.2,12)**
***Ascariasis only***				
No	85	13	1	1
Yes	91	29	2.1(1.02,4.3)	1.2(0.5,3)
***Hook worm infection***				
No	171	40	1	1
Yes	5	2	1.7(0.3,90.0)	2.4(0.3,22)
***Trichuriasis only***				
No	170	41	1	1
yes	6	1	0.7(0.1,15.9)	0.8(0.1,9)

**P value < 0.01; Multivariate analysis for underweight was done for children 7-9 years old. In the fully adjusted model: sex, age, family size, maternal education, monthly income, household food insecurity, infection with ascariasis, trichuriasis, hookworm and helmithiasis were used for underweight, stunting and wasting. For wasting in addition to these factors intakes of energy and carbohydrate were used.

Dale Woreda, Southern Ethiopia, 2012.

Age of a child (AOR = 3.4; 95% CI, 1.7-6.6), household food insecurity status (AOR = 4.1; 95% CI, 1.6-10) and maternal education (AOR = 0.3; 95% CI, 0.2-0.8) were found to be independent predictor of the height for age status. Furthermore, children with poor personal hygiene score were more likely to be stunted (AOR = 6.9; 95% CI, 2.8-17) (Table 4).

**Table 4:** **Multivariate analyses of risk factors for stunting in Southern Ethiopia**

**Variable**	**Normal Ht/age**	**Stunted**	**Crude OR**	**Adjusted OR (95% CI)**
***Sex***				
Male	156	53	1	1
Female	175	61	1(0.7,1.6)	0.9(0.6,1.7)
***Age***				
7_9	108	15	1	1
10_14	223	99	3.2(1.8,5.8)	3.4(1.7,6.6)***
***Maternal education***				
No formal education	157	78	1	1
Read and write	103	21	0.4(0.2,0.7)	0.5(0.3,0.9)*
Primary education	66	12	0.4(0.2,0.7)	0.3(0.2,0.8)**
Secondary and above	5	3	1.2(0.3,5.8)	2.5(0.2,7.0)
***Family monthly, USD***				
≤10	179	73	1	1
11-25	104	29	0.7(0.4,1.1)	0.6(0.3,1.1)
>25	48	12	0.6(0.3,1.2)	0.6(0.3,1.5)
***Ascariasis***				
No	165	57	1	1
Yes	166	57	1.0(0.6,1.5)	0.8(0.5,1.4)
***Hook worm***				
No	320	112	1	1
Yes	11	2	0.5(0.1,2.4)	0.4(0.1,2.4)
***Trichuriasis***				
No	322	104	1	1
Yes	9	10	3.1(1.4,8.7)	3.9(1.4,11.6)*
***Food security status***				
Food secure	200	42	1	1
Mild food insecure	90	39	2.1(1.2,3.4)	1.4(0.8,2.5)**
Moderate food insecure	13	16	5.9(2.6,13)	4.1(1.6,10)***
Severe food insecure	28	17	2.9(1.4,5.7)	2.5(1.0,5.6)***
***Family size***				
Small	108	28	1	1
Medium	156	52	1.3(0.7,2.1)	1.1(0.6,2.0)
Large	67	34	2.0(1.1,3.5)	1.7(0.8,3.4)
***Personal hygiene***				
Very good	45	9	1	1
Good	230	55	1.2(0.5,2.6)	1.3(0.5,3.0)
Poor	56	50	4.5(1.9,10)	6.9(2.8,17)

***P value < 0.001, **P value < 0.01, *P value <0.05.

In the fully adjusted model : sex, age, family size, maternal education, monthly income, household food insecurity,infection with ascariasis,trichuriasis, hookworm and helmithiasis were used for underweight, stunting and wasting. For wasting in addition to these factors intakes of energy and carbohydrate were used.

Dale Woreda, Southern Ethiopia, 2012.

Living in a food insecure household (AOR = 4.8; 95% CI, 1.7-13.6), inadequate intake of carbohydrate (AOR = 3.1; 95% CI, 1.4-6.8) and having a large family size (AOR = 3.3; 95% CI, 1.4-7.9) are identified as major risk factors for wasting (Table 5).

**Table 5:** **Multivariate analysis of risk factors for wasting in Southern Ethiopia**

**Variable**	**Normal BMI/age**	**Wasted**	**Crude OR(95% CI)**	**Adjusted OR (95% CI)**
***Sex***				
Male	172	37	1	1
Female	209	27	0.6(0.3,10)	0.6(0.3,1.1)
***Age***				
7_9	113	10	1	1
10_14	268	54	2.3(1.1,4.6)	2.2(1,4.8)
***Maternal education***				
No education	191	44	1	1
Read and write	112	12	0.5(0.2,0.9)	0.6(0.3,1.3)
Primary education	70	8	0.5(0.2,1.1)	0.6(0.2,1.4)
Secondary	8	0	NA	NA
***Ascariasis***				
No ascariasis	190	32	1	1
Yes ascariasis	191	32	1.0(0.6,1.7)	1.0(0.5,1.9)
***Hook worm***				
No hook worm	369	63	1	1
Yes hook worm	12	1	0.5(0.1,3.8)	0.5(0.1, 4.6)
***Trichuriasis***				
No trichuriasis	366	60	1	1
Yes trichuriasis	15	4	1.6(0.5,5.1)	2.0(0.6,7.2)
***Food security status***				
Food secure	219	23	1	1
Mild	109	20	1.8(0.9,3.3)	1.6(0.8,3.3)
Moderate	17	12	6.7(2.9,15.8)	4.8(1.7,13.6)***
Severe	36	9	2.4(1.0,5.5)	2.1(0.8,5.6)
***Family size***				
Small	128	8	1	1
Medium	170	38	3.6(1.6,7.9)	3.3(1.4,7.9)**
Large	83	18	3.5(1.4,8.3)	2.5(0.9,6.7)
***Family monthly, USD***				
<10	213	39	1	1
11-25	118	15	0.7(0.4,1.3)	0.7(0.3,1.4)
>25	50	10	1.1(0.5,2.3)	1.6(0.6,3.8)
***Personal hygiene***				
Very good	47	7	1	1
Good	249	36	0.9(0.4,2.3)	0.8(0.3,2.3)
Poor	85	21	1.6(0.6,4.2)	1.5(0.5,4.6)
***Energy intake***				
Adequate	203	25	1	1
Inadequate	178	39	1.8(1.0, 3.0)	1.3(0.7,2.4)
***CHO intake***				
Adequate	342	44	1	1
Inadequate	39	20	3.9(2.1, 7.5)	3.1(1.4,6.8)**

***P value < 0.001, **P value < 0.01. BMI = body mass index, CHO = carbohydrate. In the fully adjusted model: sex, age, family size, maternal education, monthly income, household food insecurity, infection with ascariasis,trichuriasis, hookworm and helmithiasis were used for underweight, stunting and wasting. For wasting, in addition to these factors intakes of energy and carbohydrate were used.

## Discussion

The nutritional status of children in this study showed that under-nutrition was prevalent as compared to the WHO/2007 international reference standards [10]. In this study, multiple factors are associated with under-nutrition which was similarly explained by a study done in Brazil [11]. This study found that children live in food insecure households are more likely to be stunted, underweight and wasted. Which is supported by a study done by Belachew and his colleagues [12]. Ali and his colleagues also found that severe household food insecurity was significantly associated with underweight in Ethiopia [13].

A study done by Donna and his associates among Hispanic children stated that food insecurity was negatively associated with children’s BMI for age [14]. However, a study done in USA revealed that there is no significant association between household food insecurity status and nutritional status of schoolchildren [15]. The fact that the problem of food insecurity is low in the USA may be the reason for the absence of association. Reports from different organizations like the World Bank statistics documented that, children who live in households lacking access to sufficient food are more likely to predispose to poor nutrition and health related problems than children from food secure households [16].

According to the current study, children whose mothers have never attended a formal education were more likely to be stunted and underweight as compared to children whose mothers had formal education. This is in line with a study done by Joshi and his colleagues and Emina and his associates [17,18]. Similarly, a study done in Nigeria, Pakistan and Pune revealed that low maternal education is one of the risk factor for stunting in the studied children [19-21].

In the present study, infection with Trichuris trichiura was significantly associated with stunting. This is due to chronic nature of Trichuris trichiura infection; in which it can stay in a human gut for more than three years [22,23]. Similar to this finding, other studies conducted elsewhere among schoolchildren found that infection with Trichuris trichiura is associated with stunting [24-26]. However, a study done in different areas of Ethiopia reported that there is no significant association between Trichuris trichiura infection and stunting [27-29].

In the current study it is found that as age of a child increase the likely hood of a child to be stunted will be increased this might be due to the fact that stunting is a chronic nutritional problem in which, once a child is stunted it might be difficult to revise in the late childhood. In this study, having large family size was found to be a risk factor for wasting. This is in line with a study done in North West Ethiopia [30]. Similarly, a study reported by Dona and his associates and Babar and his colleagues showed that the larger the size of family the poorer the nutritional status of the children would be seen [14,20].

According to the present study, none of the macronutrients or micronutrients were found significantly associated with under-weight or stunting. The findings are consistent with a study done in Vietnam [31]. However, a study done in Meghalaya/north east India found that the average energy intake was significantly lower in children with stunting than in children who had normal height for age status [32].

In the current study carbohydrate intake of children in the preceding 24 hours was found to be an independent factor for children’s BMI for age status. In contrary to this, a study done in Iran revealed that there was no significant association between carbohydrate intake and wasting [33]. The presence of association between carbohydrate intake and wasting in the current study may be because wasting is an acute form of malnutrition in which a recent inadequate intake of food may affect the BMI for age status. The explanation for the absence of association between dietary intake and under-weight or stunting in the current study may be, because stunting is a chronic nutritional problem. In addition to this, a single 24 hr data may not show the usual intake of participants.

### Limitation

The school based cross sectional nature of this study might lead to miss children who did not get a chance to attend school and it also limited us to not determine the direction of association. A single 24 hour recall dietary data might not reflect the usual intake of participants. This study did not assess puberty which may affect the nutritional requirement and nutritional status of studied children.

## Conclusions

In the current study, under-nutrition in the schoolchildren has multiple factors; Low maternal education and household food insecurity status were the independent factors for under-weight. Having large family size, inadequate carbohydrate intake and household food insecurity status were independent predictors for low BMI for age status. Having Trichuris trichura infection, living in a food insecure household, poor maternal education and children aged 10-14 years were risk factors for stunting.

No significant association was found between any of helminthic infections and under-weight or wasting.
